# The Abortion of Trade

**Published:** 1856-10

**Authors:** 


### *The Abortion Trade.*

There is something frightful in the perfect *sang froid* with which women call upon the physician to relieve them from the duties of a mother. They walk into the office of our most honorable practitioners, state their case with the same ease of manner which would mark their description of a headache, and appeal for assistance with as perfect assurance of their right to it.

This sentiment is almost universal in the community, and it is nothing uncommon to see a most virtuous indignation on the part of the suffering female, as she listens to the refusal of her medical adviser to take any part in such an undertaking.

We have no space or disposition to discuss the morality of this infamous trade, but we do wish to enter our protest against those practitioners who accommodate themselves to this depraved moral sense of the female. We have an indirect personal knowledge of physicians who never refuse this kind of business; and whenever we procure any positive evidence we mean to use it against them. It is not our duty to act as a detective police, but there is room in this country for action in its medical societies with reference to some of their members; and we do not doubt that when the matter is brought up in proper shape, they will inflict the proper punishment without fear of consequences.—*American Med. Gazette.*

